# Social rank affects the endocrine response to frequent regroupings in grazing dairy heifers

**DOI:** 10.3168/jdsc.2023-0494

**Published:** 2024-05-10

**Authors:** C. Fiol, M. Moratorio, M. Carriquiry, R. Ungerfeld

**Affiliations:** 1 Departamento de Producción Animal y Salud de los Sistemas Productivos, Facultad de Veterinaria, Universidad de la República, 80100, Libertad, San José, Uruguay; 2 Departamento de Producción Animal y Pasturas, Facultad de Agronomía, Universidad de la República, 12900 Montevideo, Uruguay; 3 Departamento de Biociencias, Facultad de Veterinaria, Universidad de la República, 11600 Montevideo, Uruguay

## Abstract

The objectives of this study were to determine the social behavior response to repeated social regrouping (SR), and if social rank and the changes of social rank affect the growth rate and some metabolites and hormones linked to energy status, in “resident” replacement dairy heifers subjected to SR for 205 d. Fourteen dairy heifers (153.3 ± 16.1 kg; 9 to 11 mo old), maintained under grazing conditions, were regrouped every 21 d with 5 new animals (total = 10 SR). Two days after each SR, heifers were weighed and their withers height was measured, and blood samples for nonesterified fatty acids, glucose, and IGF-1 were taken. Concentrations of nonesterified fatty acids, glucose, and IGF-1 were taken. Social behavior was registered by continuous sampling and social status was calculated by the ETlog software, considering the total number of agonistic interactions on 2 d before and 7 d after each SR. A dominance scale was constructed in each SR, and heifers were categorized as high-ranked (HRA), medium-ranked (MRA), and low-ranked (LRA). In addition, the social rank in the first and the last 5 SR were compared, resulting in 3 categories: heifers that remained in the same social rank (Mai), and heifers that lowered (Low) and raised (Rai) their social ranks. Variables were analyzed by repeated measures using a generalized lineal mixed model, considering the social rank in each SR or the change in social rank, the number of SR and their interaction as main effects, and the farm of origin and animal as random effects. Heifers performed more agonistic (2.6 vs. 1.8 ± 0.6, day of SR and 7 d later, respectively) and total (3.8 ± 1.2 vs. 2.5 ± 0.7, day of SR and 7 d later, respectively) interactions on the day of each SR than 7 d after, whereas the number of interactions decreased after the first 2 SR. Medium-ranked heifers presented greater IGF-1 concentrations than HRA heifers at SR1, SR4, and SR9, and than LRA heifers at SR3 and SR4, whereas IGF-1 concentrations were greater in HRA heifers than MRA heifers at SR3 and SR7. In addition, LRA heifers had greater IGF-1 concentrations than MRA ones at SR3 and compared with HRA heifers at SR9. Heifers that lowered their social had greater IGF-1 concentrations than Mai heifers along the SR (132.5 ± 17.1 vs. 97.8 ± 11.2 ng/mL, Low and Mai heifers, respectively) and at SR1, SR3, and SR9. In conclusion, although regrouping animals according to their characteristics can facilitate farm handling, it should be considered that endocrine profile may be affected according to heifers' position in the social hierarchy.

## Highlights

• Social regroupings triggered the display of agonistic interactions.
• The number of agonistic interactions decreased and stabilized after the first SR.
• Medium-ranked presented greater IGF-1 concentrations than high- and low-ranked ones at SR1, SR4, and SR9.
• IGF-1 concentrations were greater in heifers that lowered than in those that maintained social rank.
• Endocrine response to repeated SR depends on the individual position in the hierarchy.

## Graphical Abstract

**Figure:**
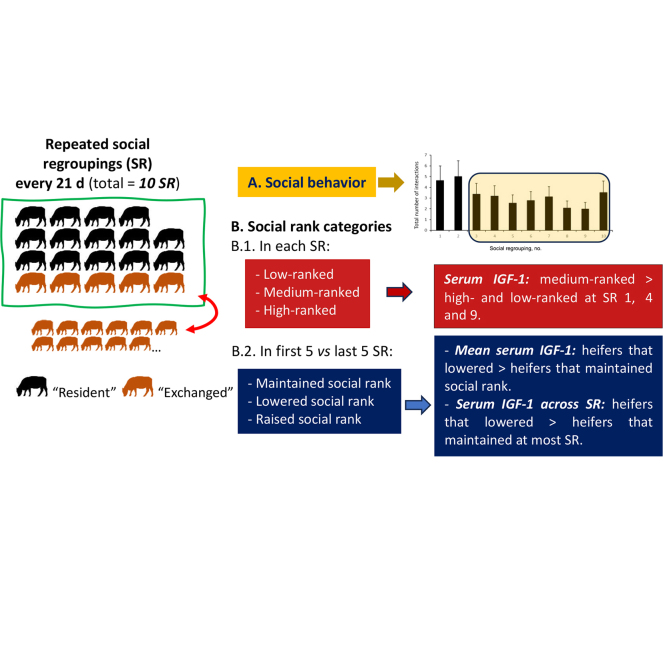
**Summary** Social regroupings (SR) are known social stressors that affect animal performance, while social rank may determine differences in response to repeated SR. We wanted to know if repeated SR influenced social behavior and if social rank and possible changes of social rank during SR determined differences in growth rate and in some metabolites and hormones associated with energy status in dairy heifers under grazing conditions. Fourteen “resident” heifers (remained in the same paddock; shown as black in the graph) were regrouped with 5 new heifers (“exchanged”; shown as red in the graph) every 21 days. The total number of interactions between resident heifers decreased and stabilized after the first 2 SR. Insulin-like growth factor 1 (IGF-1) concentrations in serum were greater in medium-ranked than in low- and high-ranked heifers in SR1, SR4, and SR9, and heifers that lowered social rank presented greater serum IGF-1 concentrations compared with heifers that maintained social rank across SR. Social rank influences the endocrine response to repeated SR in heifers.

Social relationships are involved in determining an individual's position within a group's social hierarchy (Hubbard et al., 2021a). Social position may determine changes on the productive performance (Phillips and Rind, 2002), metabolic status, and response to stressors (Chebel et al., 2016; Fiol et al., 2017, 2019) between animals within a group. Individual social positions in cattle are relatively stable over time (Kondo and Hurnik, 1990), but when animals are submitted to social regroupings (**SR**), the social hierarchy is challenged (Hasegawa et al., 1997; Raussi et al., 2005). The individual position in the hierarchy is established through agonistic interactions, and after stabilization, is mainly maintained through nonphysical agonistic interactions (Kondo and Hurnik, 1990). Social regroupings trigger an increase in the frequency of agonistic behaviors to re-establish a new hierarchical order (Hubbard et al., 2021b) and a reduction of affiliative interactions. Repeated SR affect productive performance (Hasegawa et al., 1997; Schirmann et al., 2011), metabolic status (Silva et al., 2013), and behavioral patterns (von Keyserlingk et al., 2008; Moratorio et al., 2023). Although some studies reported habituation to repeated SR, reducing the number of agonistic interactions along the successive regroupings (Veissier et al., 2001; Gupta et al., 2008), other authors reported that the aggressive interactions increase after each SR (Raussi et al., 2005; Talebi et al., 2014).

Animals may change their position in the hierarchy after each SR, and those changes may modify their coping ability and the possible consequences of SR (Hasegawa et al., 1997; Solano et al., 2004). In regrouped lactating heifers, those who lowered their individual position show a more drastic reduction in milk production than those that increase their social rank (Hasegawa et al., 1997). In contrast, milk yield persistency was negatively related to dominance rank after SR in dairy cows (Arave and Albright, 1976), and high-ranked cows were more stressed than low-ranked ones after repeated handling (Solano et al., 2004).

Social environment may determine changes in growth rates in growing heifers (Bach et al., 2006; Fiol et al., 2017). Reduced growth rates are associated with a worse energy status, characterized by a decrease of IGF-1 and glucose (Fiol et al., 2017; Rosadiuk et al., 2021), and an increase of nonesterified fatty acid (**NEFA**) concentrations (Abeni et al., 2019). In addition, greater serum NEFA concentrations are observed associated with increased serum glucose concentrations in chronically stressed animals, as those frequently regrouped (Chebel et al., 2016). The individual position in the social hierarchy may influence the energy status or stress response (or both) to regrouping, and thus may determine differences in growth rates and metabolic and endocrine profile in heifers.

We hypothesized that (1) agonistic interactions in frequently regrouped heifers increase after each SR and along the repeated SR, and (2) the growth rate and the metabolic and endocrine profiles are more affected (worse energy status: lower growth rates, glucose, and IGF-1; and greater NEFA) by repeated SR in low than in high-ranked heifers and in those heifers that lowered their social rank compared with those that maintained or raised their social rank after SR. We aimed to determine the social behavior response to SR, and if social rank and changes of social rank affect growth rates and metabolic and endocrine profile, in “resident” replacement dairy heifers subjected to repeated SR.

Animal care, handling, and protocols were approved by the Ethics Commission on the Use of Animals (number 465 CEUA, Universidad de la República, Uruguay). The study was performed on a private contract-rearing farm (Uruguay, 34°S, 55°W) from September (early spring) to May (autumn). The general facilities and procedures were described in a previous study, as both were performed with the same animals (Moratorio et al., 2023), but this study focused on the response of the regrouped heifers. Fourteen Holstein heifers (153.3 ± 16.1 kg; 7 to 10 mo old) remained in the same grazing paddock with another 5 Holstein heifers that were exchanged every 21 d (total = 210 d), subjecting the basic group (“resident” heifers) to frequent SR. On the day of each SR, between 0800 and 1000 h (total = 10 SR), 5 unknown heifers (with no previous contact with the resident ones) were introduced at the time that the previous 5 were retired from the group. Those 5 new heifers were of similar BW (182.3 ± 5.2 kg) and age (range = 1 mo) compared with the 14 resident heifers, and were selected from the general herd, totaling 64 heifers participating in the study. The heifers grazed a mixed pasture of fescue and red clover on weekly stripes (forage allowance = 8 kg DM/100 kg BW) throughout all the study and had free access to cutwaters. Pasture availability was monitored monthly (range: 810 to 2,930 kg DM/ha; Haydock and Shaw, 1975). The size of the paddock ranged from 0.8 to 2 ha (minimum of 420 m^2^ per heifer), depending on pasture availability. Two days after each SR (0700 to 1000 h), heifers' BW and withers height (**WH**) were measured and individual ADG were calculated to determine body development (Heinrichs et al., 1992, 2017). On SR6 it was not possible to determine BW nor ADG; thus, data for those variables are only available in 9 of the 10SR. Blood samples were collected simultaneously with body development measurements, and samples were centrifuged and stored at −20°C until measurement of NEFA, glucose, and IGF-1 concentrations (Moratorio et al., 2023). Samples for IGF-1 concentrations were only analyzed on 6 of the 10 SR (SR1, SR3, SR4, SR5, SR7, and SR9).

Social behavior was registered by continuous sampling 2 d before each SR, the day of SR, and 7 d later, during the last 3 h of daylight (1700 to 2000 h). During the study, a total of 4 trained observers recorded, by direct visual observation, and categorized the different social interactions between the resident heifers: head butting (pushing another with its head on any part of the body), chasing (chasing another with no physical contact), displacing (displacing another by physical contact, withdrawal of the reactor), and threatening (displacing another with no physical contact, the reactor may or may not withdraw), whereas affiliative interactions were mounting (mounting or trying to mount another), licking (licking another, in any part of the body), and scratching (scratching any part of the body toward another animal). To assess the interobserver agreement, the 4 observers conducted a simultaneous 2-d preliminary observation assessment on the same animals, during which they recorded the different behaviors for two 1-h periods each day (overall agreement = 0.9 for each behavior; data not shown). A big number was painted on the back of each heifer to allow identification from long distances. Both the animal that initiated each interaction and the one that received it were recorded. To correct possible biases, the observers were blocked and assigned to each day. As the aim was to evaluate the effects of regrouping on the resident heifers and not on the “exchanging” ones, all responses were only measured in the 14 resident heifers.

Social status was determined by the ETlog software (Deniz, 2018), performed in R (R Core Team, 2020). The ETlog considered all agonistic interactions registered 2 d before and 7 d after each SR in which an animal was involved (i.e., as actor or reactor) in relation to another herd member, of each possible dyad of the group of resident heifers. Days were chosen to minimize the acute effects of SR on social hierarchy (Kondo and Hurnik, 1990). In each SR, the dyadic dominance relationship of the *i*th animal relative to the *j*th animal (*Sij*) is assessed qualitatively by the sign of the difference between *X_ij_* and *X_ji_*, as proposed by Kondo and Hurnik (1990): $S_{ij}=\frac{X_{ij}-X_{ji}}{\left|X_{ij}-X_{ji}\right|},$ where *S* is the relation between animal *i* and animal *j*; *Xij* is the number of winning interactions of *i* animal over *j* animal and *X_ji_* is the number of winning interactions of *j* animal over *i* animal, which always results in a value of −1 (animal *i* had less winning interactions than animal *j*), 0 (animal *i* had the same number of winning interactions as animal *j*), or +1 (animal *i* had more number of winning interactions than animal *j*) (Kondo and Hurnik, 1990). With the obtained results of the previous equation, the dominance value for each individual was calculated according to $S_{i}=\overset{n}{\underset{j-i}{\sum }}\;S_{ij},$ where *S_i_* is the sum of all the interactions in which *i* animal was involved, and *n* is the number of possible interactions of one animal of the group with the others. Dominance position was then assigned according to *S_i_* values, from highest (α) to lowest (ω). When 2 animals had the same *S_i_* value, the tiebreaker was the result of the dominance relationship among the dyad. With the sociometric matrix, the ETlog software calculated the linearity index proposed by Landau (1951) and applied the improved test of linearity (h') due to the unknown relationships as described by De Vries (1995). Finally, a dominance scale was constructed in each SR, based on the difference between the maximum and minimum dominance values plus 1 (corresponds to the dominance value zero), and heifers were categorized in 3 social ranks: heifers in the first tertile were classified as low-ranked (**LRA**), the ones in the second tertile as medium-ranked (**MRA**), and heifers in the third tertile (higher positive dominance values) of the dominance scale were classified as high-ranked (**HRA**; data not shown).

All analyses were performed with SAS on Demand for Academics (SAS Institute Inc.). The normal distribution of all data and residues was checked with the UNIVARIATE procedure. The mean number of social interactions (not normally distributed) was analyzed with a generalized linear mixed-effects model using the GLIMMIX procedure (Poisson distribution):

*Y_ij_* = *μ* + *T_i_* + *H_j_* + (*T*×*H*)*_ij_* + *B_i_* + *C_j_* + *e_ijk_*,

where *Y_ij_* was the dependent variable (agonistic, affiliative, and total interactions), *μ* the general mean, *T_i_* was the fixed effect of the SR (*i* = SR1 to SR10), *H_j_* the fixed effect of the day of social behavior determination (*j* = d 0 vs. 7), (*T*×*H*)*_ij_* the interaction between *T* and *H*, *B_i_* the random effect of the animal, *C_j_* the random effect of the farm of origin, and *e_ijk_* the residual error.

The effect of the individual position in the hierarchy on growth rate and metabolic and endocrine profiles was analyzed as a repeated measure according to social rank in each SR (LRA, MRA, and HRA heifers). In addition, to determine the effects of social rank changes across the SR, the social rank in the first and the last 5 SR were compared, resulting in 3 categories: heifers that remained in the same social rank (**Mai**), heifers that lowered their social rank (**Low**) and heifers that raised their social rank (**Rai**; data not shown). For this, as the social rank was stable along the first 5 and along the last 5 SR, the social rank of the first 5 and last 5 SR was considered as that in which the heifer was categorized more times (4 of 5 or 3 of 5 SR). For example, heifer number 1 in the first 5 SR was classified as LRA, LRA, LRA, MRA, LRA; thus, the social rank of the heifer on the first 5 SR was LRA. For the last 5 SR she was classified as LRA, MRA, LRA, MRA, LRA; thus, the social rank for the last 5 SR was LRA. Then, heifer number 1 was defined as Mai.

The effects of social rank in each SR and social rank changes across the first 5 and last 5 SR on body growth parameters and metabolite concentrations were analyzed by a linear mixed-effects model, as follows:

*Y_ijk_* = *μ* + *T_i_* + *A_j_* + *H_k_* + (*T*×*H*)*_ik_* + (*A*×*H*)*_jk_* + *B_i_* + *C_j_* + *e_ijkl_*,

where *Y_ijk_* was the dependent variable (BW, ADG, WH, and serum NEFA, glucose, and IGF-1 concentrations), *μ* the general mean, *T_i_* was the fixed effect of social rank (*i* = HRA, MRA, and LRA), *A_j_* the fixed effect of social rank change (*j* = Low, Mai, and Rai), *H_k_* the fixed effect of the SR (repeated measure; *k* = 1 to 10), (*T*×*H*)*_ik_* the interaction between social rank and SR, (*A*×*H*)*_jk_* the interaction between social rank changes and SR, *B_i_* the random effect of the animal, *C_j_* the random effect of the farm of origin, and *e_ijkl_* the residual error. The covariance structure AR (1) (for constant periods) or SP (pow) (for nonconstant periods) were used. Body weight, ADG, WH, serum NEFA, and glucose were analyzed by the MIXED procedure, whereas IGF-1 concentrations (Poisson distribution) were analyzed using the GLIMMIX procedure. Baseline BW and concentrations of NEFA, glucose, and IGF-1 were included as covariates in the corresponding analyses. In both GLIMMIX and MIXED procedures, the Kenward-Rogers method was used to adjust denominator of degrees of freedom, and Tukey-Kramer tests were conducted to analyze differences between groups. Differences were considered significant when *P* ≤ 0.05 and as tendencies when 0.05 < *P* ≤ 0.1. Results are presented as LSM ± SEM.

Overall, 763 (509 agonistic and 254 affiliative) social interactions were registered. Heifers performed more agonistic (2.6 vs. 1.8 ± 0.6, day of SR and 7 d later, respectively) and total (3.8 ± 1.2 vs. 2.5 ± 0.7, day of SR and 7 d later, respectively) interactions on the day of SR than 7 d later (*P* = 0.01 for both comparisons), with no differences in affiliative interactions (2.1 ± 0.6). In addition, social interactions changed along the different SR; in general, the number of interactions decreased throughout the study in relation to the first 2 SR (Figure 1).

**Figure 1:**
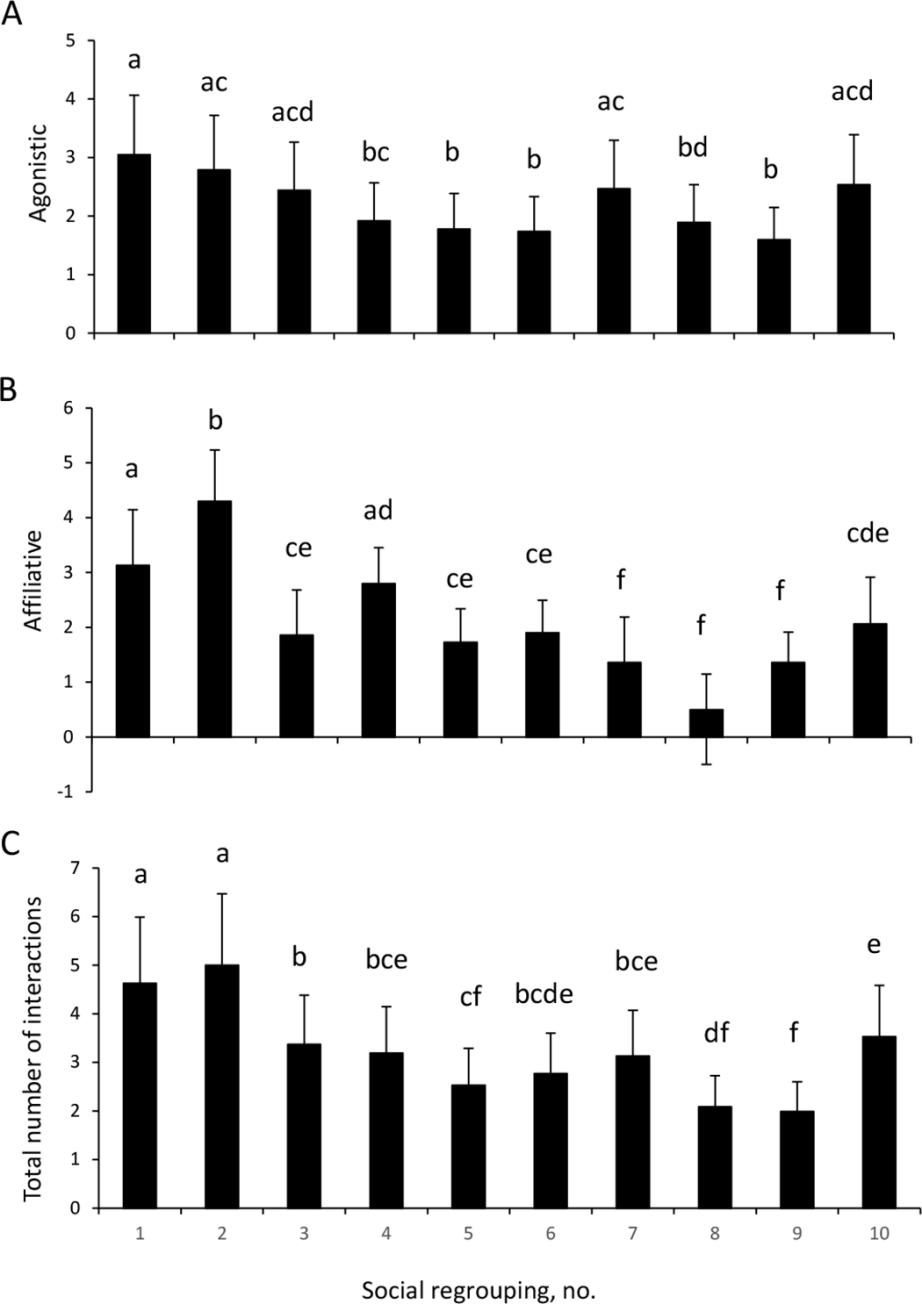
Mean number of agonistic (A), affiliative (B), and total (agonistic + affiliative; C) interactions in dairy replacement heifers (n = 14) regrouped every 21 d with 5 new heifers for 205 d, according to the number of social regrouping (SR). Data were analyzed according to the number of SR (SR1 to SR10) using the GLIMMIX procedure. Time (number of SR) was considered the main effect, and the animal and farm of origin were considered the random effects. Tukey-Kramer tests were conducted to analyze differences between SR. Different letters in each column represent significant differences (*P* < 0.05) between SR. Error bars represent SEM.

Mean BW was not related to heifers' social rank in each SR (258.5 ± 14.3 kg), but it tended to change across SR according to the heifers' social rank (*P* = 0.08). No social rank effect nor interaction of social rank by SR was found for ADG (0.780 ± 0.05 kg/d), WH (117.9 ± 2.9 cm), glycemia (4.2 ± 0.4 mmol/L), or NEFA concentration (0.4 mmol/L), whereas WH increased along the SR (*P* = 0.01). Mean IGF-1 concentrations were similar (*P* = 0.2) between heifers of different social rank (109.0 ± 15.0 ng/mL), but there was an interaction of social rank by SR (*P* = 0.03), as IGF-1 concentrations were higher in HRA (*P* = 0.03) than MRA heifers at SR3 and SR7 (Figure 2A). In addition, IGF-1 concentrations were greater in MRA than HRA heifers at SR1, SR4, and SR9, and compared with LRA heifers at SR1 and SR4 (*P* = 0.01), whereas LRA heifers had greater IGF-1 concentrations than MRA ones at SR3 and compared with HRA heifers at SR9 (*P* = 0.01; Figure 2A).

**Figure 2:**
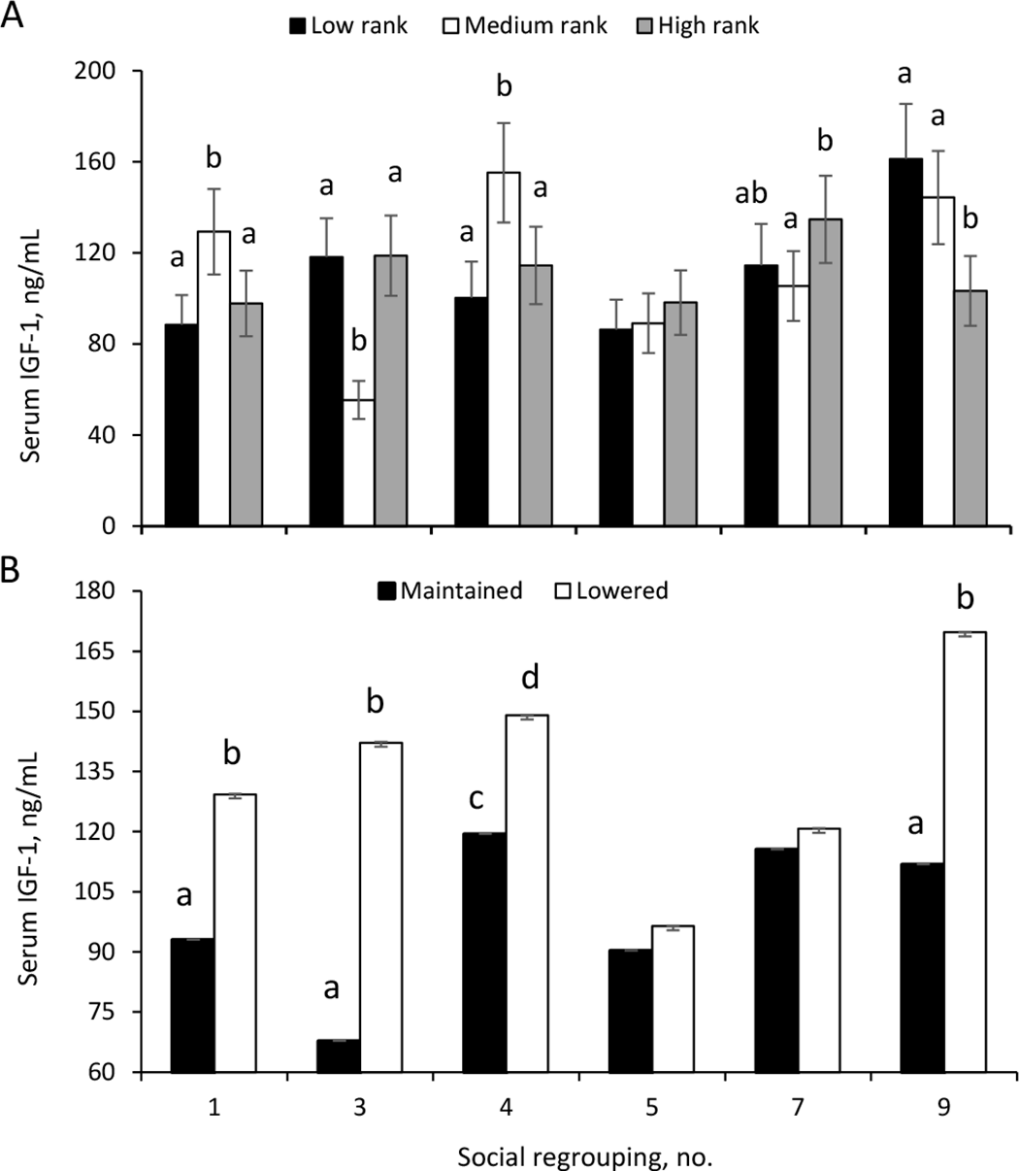
Serum IGF-1 concentrations in dairy replacement heifers (n = 14) regrouped every 21 d with 5 new heifers according to the social rank (panel A) and to changes in social rank (panel B) in each social regrouping (SR). Social rank was determined according to agonistic interactions in each SR (ETlog software; Deniz, 2018), and heifers were categorized as low, medium, or high social rank. In addition, heifers were categorized as those that maintained (Mai) or lowered (Low) their social rank in the last 5 SR compared with the first 5 SR. Panel A: different letters represent significant differences (*P* < 0.05) between heifers in each SR. Panel B: different letters represent significant differences between Low and Mai heifers in each SR: a vs. b = *P* < 0.05; c vs. d = *P* ≥ 0.05 ≤ 0.1. Error bars represent SEM.

Concerning social rank changes, 7 animals maintained their rank, 5 lowered, and 2 raised their social rank. Considering the low number of animals that raised their social rank, growth rates, metabolites, and IGF-1 analysis were performed comparing only Mai and Low heifers. Mean ADG were similar between Low and Mai heifers throughout the study (0.800 ± 0.04 kg/d), but there was an interaction between the changes in social rank and SR for ADG, as in SR4 Low heifers gained more BW than Mai heifers (0.99 ± 0.13 vs. 0.61 ± 0.11 kg/d, Low and Mai heifers, respectively; *P* = 0.03). In addition, IGF-1 concentrations were greater in Low than Mai heifers along the SR (132.5 ± 17.1 vs. 97.8 ± 11.2 ng/mL, Low and Mai heifers, respectively; *P* = 0.04). There was also an interaction between the change in social rank and SR for IGF-1 concentrations (*P* = 0.08); Low heifers had greater IGF-1 concentrations at SR1, SR3, and SR9, and tended to be greater at SR4, than Mai heifers (Figure 2B). There were no effects of change of social rank nor of interaction social rank by SR in any other variable analyzed (data not shown).

As hypothesized, SR triggered an increase in the display of agonistic interactions, but the frequency of agonistic interactions decreased after the first 2 SR and remained stable along the rest of the study. This suggest that at least, even when exposed to a chronic repeated stressor, heifers could habituate and cope with social instability. In addition, not all heifers could adapt similarly, as the evolution of IGF-1 concentrations was related to hierarchical positions and to the changes in individual social positions that heifers experienced along the repeated SR. Age is a factor related to social hierarchy (Hubbard et al., 2021a), so it would have been important to have the exact age of each animal, but because they came from different commercial farms, it was not possible to obtain all the exact data, limiting the consideration of these data in the study.

As previously reported (Raussi et al., 2005; von Keyserlingk et al., 2008), regrouping disturbs social behavior, as heifers display more agonistic interactions immediately after each SR than 1 wk later. However, as the number of SR progressed, the frequency of agonistic interactions remained stable. In contrast, the number of agonistic interactions of the introduced animals increased with the successive regroupings, which was related to the lack of habituation of the animals to frequent SR (Raussi et al., 2005). Introduced individuals, as opposed to resident ones, must deal with SR simultaneously with the challenge of a new allocation and general facilities. According to our knowledge, few studies have evaluated the effects of SR on the animals that remain as residents (Bach et al., 2006; Rocha et al., 2020). Rocha et al. (2020) reported that social bonds in a group of resident animals get stronger over time and fewer aggressive interactions are necessary to re-establish hierarchy after regrouping with new animals. In that sense, resident heifers in this study could develop coping strategies that imply no increases in their aggressiveness, probably redirecting the aggressive requirements to the group of introduced heifers. Finally, it should also be considered that heifers remained grazing extensively, with a low stocking density (minimum of 420 m^2^ per heifer), reducing the negative effects of SR because animals have enough space to avoid contact between them, and thus reducing the number of possible aggressive encounters (Talebi et al., 2014).

We found differences in the endocrine profile according to social rank: IGF-1 concentrations were greater in MRA than in HRA and LRA heifers at SR1, SR4, and SR9. In contrast, we expected to find increase IGF-1 concentrations in heifers of higher social ranks, possibly associated with a lower level of social stress in heifers on the upper stratum of the hierarchy compared with the ones in the medium and low strata. In that sense, previous studies reported that low-ranked cows presented greater cortisol concentrations after SR (Mench et al., 1990), and more negative effects on milk production in subordinate dairy cows after SR (Hasegawa et al., 1997; Soonberg et al., 2021), compared with animals of high and medium social rank. It is necessary to consider that the low number of animals evaluated may limit detecting some other possible effects. In agreement with our findings, Miranda-de la Lama et al. (2013) reported that medium-ranked males had greater ADG, lower cortisol concentrations, and better meat quality than high- and low-ranked bulls. Possible explanations to the contrasting results are not obvious; it may be argued that high-ranking heifers are less successful in coping with SR in comparison to medium-ranking ones (Arave and Albright, 1976; Chebel et al., 2016), or that they are more sensitive to the negative consequences of social stress than medium-ranked ones (Miranda-de la Lama et al., 2013).

The effects of changes in social position along the SR were unexpected, as heifers that lowered their social rank had greater serum IGF-1 concentrations along the study and at SR1, SR3, and SR9 than those heifers that maintained their social rank. High growth rates were associated with an increase of serum IGF-1 (Rosadiuk et al., 2021), whereas changes in social rank status were expected to be linked to the social stress response to SR (Chebel et al., 2016). Thus, the greater IGF-1 concentrations in heifers that lowered their social position after SR may be related to reduced levels of social stress or changes in feeding utilization compared with those heifers that successfully maintained their social rank across SR. As previously mentioned, the low number of animals evaluated may limit detecting some possible effects.

In conclusion, agonistic interactions increased on the day of each SR, but this response was decreasing along the successive SR. Social rank and social rank changes were associated with the endocrine profile in heifers subjected to repeated SR, as serum IGF-1 concentrations were greater in medium-rank heifers than in high and low-ranked ones, and in those heifers that lowered their social rank compared with those that maintained their social rank. Thus, individual position in the social hierarchy may influence the endocrine response to SR in the farm.
